# Ultrasonically synthesized Cu/Ag/Zn nanocomposite coatings for antibacterial and antibiofilm applications in urological devices^☆^

**DOI:** 10.1016/j.ultsonch.2025.107674

**Published:** 2025-11-11

**Authors:** Houhong Wang, Chun Luo, Wenli Chen, Weilin Wang, Yongyun Chen, Kelei Shang, Yan Peng, Zhongmin Li

**Affiliations:** a Department of General Surgery, The Affiliated first Hospital of Fuyang Normal University, China; b Department of Paediatric Surgery, The Affiliated first Hospital of Fuyang Normal University, China; c Department of General Surgery, The Affiliated Bozhou Hospital of Anhui Medical University, China; d Fuyang Normal University Fuyang Medical College, China; e Department of Ophthalmology, The Affiliated first Hospital of Fuyang Normal University, China

**Keywords:** Cu/Ag/Zn trimetallic nanocomposites, Urinary catheter coating, Antibacterial activity, Anti-biofilm, Ultrasonic-assisted synthesis, Surface engineering

## Abstract

Catheter-associated urinary tract infections (CAUTIs) pose a persistent clinical challenge due to bacterial colonization and biofilm formation on indwelling urological devices. This study investigates the efficacy of Cu/Ag/Zn trimetallic nanocomposites (TMNCs) as antimicrobial coatings for urinary catheters, synthesized via a green, ultrasonic–autoclave-assisted method. The nanocomposites were thoroughly characterized using X-ray diffraction (XRD), X-ray photoelectron spectroscopy (XPS), Fourier-transform infrared spectroscopy (FTIR), Raman spectroscopy, thermogravimetric analysis (TGA), Brunauer–Emmett–Teller (BET) surface area analysis, and field emission scanning electron microscopy (FESEM) to confirm their mesoporous, crystalline, uniformly distributed, and thermally stable nanoparticles. An optimized ultrasonic embedding technique was employed to uniformly coat TMNCs onto commercial urinary catheters, ensuring strong adhesion and consistent coverage. Antibacterial assays demonstrated concentration-dependent inhibition zones, reaching up to 21.5 mm for *Escherichia coli* and 24.0 mm for *Staphylococcus aureus*. Minimum inhibitory concentration (MIC) tests confirmed potent bactericidal activity, with MIC values of 32 µg/mL for *E. coli* and 64 µg/mL for *S. aureus*. Anti-biofilm assessments revealed that TMNCs matched or outperformed the efficacy of vancomycin, achieving up to 86.12 % inhibition for *S. aureus* and 73.59 % for *E. coli* at 2 × MIC. Cytotoxicity testing using U87 glioblastoma cells indicated good biocompatibility, with over 79 % cell viability at 0.1 mg/mL, followed by a dose-dependent decline at higher concentrations. Long-term stability studies conducted over 60 days under varied storage conditions confirmed the photothermal and colloidal stability of the TMNCs. In conclusion, Cu/Ag/Zn TMNC-coated urinary catheters demonstrated excellent antibacterial and anti-biofilm properties, biocompatibility, and long-term stability. These multi-functional coatings present a promising strategy for reducing CAUTI incidence and improving patient safety and clinical outcomes in urological applications.

## 1 Introduction

Urinary tract infections accounted for approximately 40 % of nosocomial infections, which are now one of the leading causes of death and morbidity. Of these, up to 80 % were linked to indwelling catheters, particularly catheter-related urinary tract infections (CRUTIs) [1]. A major problem in contemporary clinical treatment is catheter-associated infections, especially when it comes to long-term device placement. For the treatment of patients with long-term illnesses such as end-stage renal disease, urine retention, and different neurological abnormalities, urinary catheters are essential [2]. Even though these catheters are extremely useful, they are very prone to microbial colonisation and biofilm formation. This can result in major problems like bloodstream infections, catheter-associated urinary tract infections (CAUTIs), encrustation, inflammation, and eventually catheter failure [3].

Due to their well-known resistance to common antibiotics and disinfectants, biofilms structured colonies of bacteria embedded inside a self-produced polymeric matrix seriously jeopardise patient safety and raise healthcare expenses. Urinary catheters used to administer medication to patients need to be handled and sterilised carefully to avoid serious renal infections [4]. These illnesses may deteriorate the patient's health, resulting in bacteremia, extended hospital stays, and higher expenses. Their significant activity against both Gram-positive and Gram-negative bacteria, as well as their varied modes of action that do not result in AMR, make the latter an important alternative to antibiotics. Most importantly, mixing metals allows for the creation of new qualities or enhances those that are already present. For example, whilst gold nanoparticles (Au NPs) are thought to be biocompatible and anti-inflammatory, silver nanoparticles (Ag NPs) have potent antibacterial activity and cytotoxicity [5].

Their combined action results in decreased toxicity, more stability, and a wider surface area, which enhances the antibacterial activity. Developing new materials and surface modification techniques to counteract microbial adhesion and growth has gained attention due to the ongoing issue of biofilm-associated catheter infections [6,7]. Nanotechnology is one of these that presents a viable path toward innovation. Unique physicochemical characteristics of nanoparticles, especially those made of metals and metal oxides, include a high surface-area-to-volume ratio, increased reactivity, and adjustable surface functions, all of which improve their antibacterial efficacy [8]. Trimetallic nanocomposites are becoming next-generation antibacterial agents in this context because of their multi-mechanism action on microbial cells and synergistic qualities [9].

**Fig. 1:**
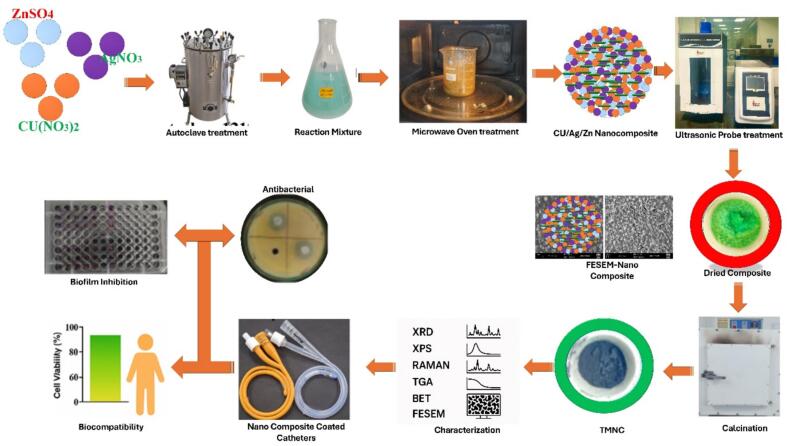
Schematic Illustration of Cu/Ag/Zn Trimetallic Nanocomposite-Coated Urinary Catheter.

In this work, we investigate the formation, properties, and antibacterial use of a Cu/Ag/Zn trimetallic nanocomposite as a powerful coating substance for urinary products (Fig. 1). These TMNCs seek to lower the prevalence of CAUTIs by inhibiting bacterial colonization and biofilm development [10]. A green ultrasonic-assisted approach was used to create the nanoparticles, which have benefits like improved reaction rates, consistent particle dispersion, and exact control over size and shape. The formed nanocomposites were tested using commercially available Foley catheters for cytotoxicity, antibacterial effectiveness, and antibiofilm potential in both static and dynamic circumstances. By combining the distinct antibacterial properties of copper (Cu), silver (Ag), and Zinc (Zn), the nanocomposite's bactericidal and antibiofilm activity is enhanced [11,12].

These metals exhibit complementary antibacterial mechanisms, including rupture of microbial cell membranes and generation of reactive oxygen species (ROS). They also cause protein denaturation and DNA damage, leading to cell death [13]. These mechanisms, when integrated in nanoscale form, limit resistance development, biofilm formation, and microbe survival simultaneously [14]. The nanocomposite was created via co-reduction and then hydrothermally treated with autoclave assistance to enhance stability, dispersion, and crystallinity. Subsequently, the formulation underwent ultrasonic-assisted processing to improve homogeneity and decrease agglomeration, allowing for effective coating. Energy-dispersive X-ray spectroscopy (EDS), dynamic light scattering (DLS), UV–Vis spectroscopy, scanning electron microscopy (SEM), zeta potential analysis, and other characterization methods were used to verify particle formation, ascertain size distribution, appraise surface morphology, and assess charge stability [[15], [16], [17]].

These methods allowed a thorough understanding of the nanocomposite's chemical and physical characteristics. To assess the antibacterial and antibiofilm effectiveness of the Cu/Ag/Zn nanocomposite, *Staphylococcus aureus* and *Escherichia coli*, known antibiotic-resistant organisms with clinical relevance in catheter-related infections, were utilized [18]. To evaluate the nanocomposite's clinical potential, minimum inhibitory concentration (MIC) tests, colony count reduction, and biofilm inhibition investigations were done. In addition, fibroblast cell lines were used in cytotoxicity tests to confirm biocompatibility and establish the safe concentration threshold for catheter coating applications [19,20]. Unlike previously reported Pb/Ag/Cu or Ag/ZnO composites, the present Cu/Ag/Zn trimetallic nanocomposite uniquely combines the redox-active Cu and Ag species with the biocompatible Zn matrix, enabling synergistic antibacterial, antibiofilm, and anti-adhesive effects. Its use as a nanocoating for urinary catheters specifically addresses CAUTI prevention by providing long-term antimicrobial protection without antibiotic leaching [21].

## 2 Materials and methods

### 2.1 Materials

The zinc sulfate [ZnSO_4_], copper (II) nitrate [Cu (NO_3_)_2_], and silver nitrate [AgNO_3_] purchased from Hi-media were ready to use without any additional purification steps. Throughout the trials, solvents such as ethanol and distilled water were utilized. For the antibacterial and antibiofilm tests, clinical strains from the Rela Hospital and Diagnostic Centre, including *Escherichia coli* and *Staphylococcus aureus*, were obtained. Cytotoxicity was assessed using the MTT test on fibroblast cell lines. Dimethyl sulfoxide (DMSO), crystal violet, phosphate-buffered saline (PBS), and MTT dye were additional reagents. Every assay was carried out with common laboratory apparatus.

### 2.2 Hydrothermal-autoclave synthesis of Cu/Ag/Zn trimetallic nanocomposites

A hydrothermal autoclave was used to create Zn/Ag/Cu trimetallic nanocomposites; this process gave the reaction a controlled environment. With this technique, the precipitation and development of nanoparticles are precisely controlled in a sealed aqueous environment at high temperatures and high pressures. As the system functions at extreme pressure and temperature, this enables customisation of the nanoparticles' size, shape, and even internal crystal structure [22,23]. The colorless purity of 0.01 M solutions of zinc sulfate, silver nitrate, and copper (II) nitrate merged after 30 min of continuous magnetic stirring. The mixed metal ion solution was transferred into a Teflon-lined stainless-steel autoclave, filled to approximately 70–80 % of its volume to allow for safe pressure buildup during the hydrothermal process.

After hydrothermal treatment, the mixture was subjected to a series of controlled procedures, including a 15-min microwave burst, a 30-min probe sonication bath with its distinctive vibrations, and another microwave treatment cycle to improve particle dispersion and decrease solvent volume. There was a slight chemical odour in the air when the solution naturally cooled to room temperature. After centrifugation, the precipitate a fine white powder was collected and repeatedly cleaned with ethanol and distilled water, which produced a swirling liquid vortex, to get rid of any remaining contaminants. It was then dried at 60 °C in a hot air oven. The powder was dried and then placed in alumina crucibles, which were heated in a muffle furnace to 900 °C for two hours, resulting in the formation of a dark Cu/Ag/Zn composite powder. A dark Zn/Ag/Cu trimetallic nanocomposite was produced and kept in sterile glass vials for additional biological analysis and characterisation [24,25].

### 2.3 Characterisation of nanocomposites

Field emission scanning electron microscopy (FE-SEM, ZEISS) equipped with an In Lens detector was used to analyse the structural morphology of the produced Cu/Ag/Zn nanocomposites. A small layer of gold was sputter-coated onto the samples to improve conductivity. Imaging was carried out using a working distance of 3.5 mm, an accelerating voltage of 5.21 kV, and magnifications of 75,000 × and 50,000 × . Uniformly distributed nanoparticles, some agglomerates, and rod-like structures were visible in the FSEM pictures [26]. The Cu/Ag/Zn TMNC's FTIR spectra were captured in attenuated total reflectance (ATR) mode using a Thermo Fisher Scientific, USA, Nicolet Summit LITE FTIR spectrometer; the device accurately assessed the amount of infrared light that the sample absorbed. The spectral data were obtained at 16 cm^−1^ intervals between 4000 and 500 cm^−1^. The investigation used to clearly identify surface functional groups, providing information about complicated chemical interactions between various metal components, potentially including [e.g., ligand exchange]. To ascertain the Cu/Ag/Zn TMNC's crystalline structure and phase composition, XRD analysis was employed [26].

The observations were made using a Philips powder diffractometer operating at 40 kV and 30 mA, using Cu Kα radiation (λ = 1.54060 Å). Accurate measurements were made possible by the modest, purposeful scanning rate of 2°/min throughout a 2θ range of 10° to 80°. Phase purity and a slight lattice strain were revealed by the sample's crisp diffraction rings, which are a sign of high crystallinity [27]. By using XPS to ascertain the constituent metals' oxidation states and surface elemental composition, comprehensive information regarding the chemical states of the constituent elements was obtained. The measurements were carried out on an Omicron ESCA (Oxford Instruments) system using Al-Kα radiation (1486.6 eV).

To take surface charging into consideration, the binding energies were calibrated by comparing them to the C 1 s peak at 284.6 eV. Based on the detected spectrum shifts, the research provided accurate information on the electronic environments and chemical states of Cu, Ag, and Zn in the TMNC, highlighting the unique electron configurations of each metal. Using a Field Emission Scanning Electron Microscope (FESEM, Zeiss SUPRA 55 Sapphire, Germany), the Cu/Ag/Zn TMNC was examined for surface morphology and elemental mapping. With the use of a Quantachrome Nova 2000e (Brunauer–Emmett–Teller) analyzer, which accurately evaluated the sample's gas adsorption, surface area and porosity values were acquired. Using nitrogen adsorption–desorption isotherms, the TMNC's specific surface area, average pore diameter, and total pore volume all crucial parameters for catalysis and adsorption—were determined. To evaluate the Cu/Ag/Zn TMNC's thermal stability, a Mettler Toledo TGA/SDTA851e thermogravimetric analyser, which measures weight changes precisely under regulated conditions, was used. As 10 mg of the material was exposed to a regulated temperature gradient from 100 °C to 900 °C at 10 °C/min, a controlled environment was maintained. With weight loss from oxidation, organic matter breakdown, and moisture evaporation all discernible as separate steps in the thermal profile, the thermogram amply demonstrated the material's thermal breakdown [28]. To assess the stability of Cu/Ag/Zn nanocomposites, 1  mL aliquots were stored for 60 days under three conditions: dark and cold (4 °C), light and room temperature, and dark and room temperature. Changes were monitored in the samples regularly. Surface plasmon resonance (SPR) peak shifts were observed by scanning the samples using UV–vis spectroscopy (300–600 nm) and periodically checking them for visual changes [29].

### 2.4 Development and optimisation of nanocomposite-coated urinary catheters

The synthesized Cu/Ag/Zn trimetallic nanocomposite (TMNC) was incorporated onto a sterile urinary catheter via an ultrasonic technique to determine its efficacy against bacteria and biofilms; the process resulted in a tightly bound, uniform film. The embedding protocol was optimized by carefully adjusting the metal concentration, ultrasonic power, and Tri metal treatment duration’s coating to ensure a uniform coating and maximum bioactivity; this involved fine-tuning each parameter to achieve the ideal balance. A preliminary dispersion was achieved by vortexing a mixture of 0.1 mg TMNC in 1 mL Milli-Q® water for 30 min, resulting in a visibly homogenous solution. Next, the solution was sonicated in an ultrasonic bath, causing the nanoparticles to evenly distribute throughout the liquid, a process noticeable by the gentle bubbling. Using an ultrasonic probe that emitted a high-pitched whine, the creamy TMNC emulsion was meticulously applied to the catheters for impregnation [30,31].

Sub-IC50 concentrations of TMNC were applied to catheters using a probe sonicator (30–90 kHz), resulting in a smooth, uniform coating; the process generated a noticeable hum. The TMNC dispersions were prepared with varying concentrations (20, 40, 60, 80, 100, 120, and 140 µL of the 0.1 mg/mL solution) to be used for embedding. To assess the impact of sonication duration on nanoparticle adhesion and coating uniformity, each concentration underwent testing with ultrasonic was performed in a bath sonicator operating at 90 kHz and 150 W for 15 min to ensure uniform nanoparticle dispersion and coating adhesion.

The urinary catheters were immersed in the TMNC dispersions, and the ultrasonic probe was used to treat them with controlled frequency and amplitude settings. Following embedding, the catheters were meticulously rinsed with sterile saline to remove any loosely bound particles, then carefully dried in a laminar flow hood. Antibacterial effectiveness against target pathogens was used to judge each condition; this functional metric determined which embedding conditions were optimal.

Additionally, the embedded TMNC layers were examined morphologically using field-emission scanning electron microscopy (FESEM). This visualisation revealed the extent of surface coverage, the uniformity of the coating, and any potential clumping of nanoparticles. A thorough examination of the optimised samples' nanoparticle embedding involved comparing them to uncoated controls and those treated pre- and post-wash. The goal was to determine the long-term stability and resistance to various conditions [32].

This multi-parameter optimisation approach revealed the ideal TMNC concentration and ultrasonic treatment duration for producing a consistent, adherent, bioactive nanocomposite coating; the resulting coating felt smooth to the touch and displayed a uniform, microscopically-verified structure.

### 2.5 Antibacterial assay

The antibacterial properties of the Ag/Cu/Zn trimetallic nanocomposite (TMNC) coating on urinary catheters were assessed using the well diffusion method and direct contact with agar plates containing *Staphylococcus aureus* (Gram-positive) and *Escherichia coli* (Gram-negative), common UTI pathogens. The tests aimed to determine the effectiveness of the coating against these bacteria. The growth medium for all antibacterial assays was Mueller-Hinton Agar (MHA), a standard medium that provides optimal bacterial growth conditions. Overnight bacterial cultures, swirling with a milky consistency matching the 0.5 McFarland standard, were carefully spread across the MHA surface. In the well diffusion assay setup, the agar was punctured with wells measuring 8  mm in diameter using a sterile borer. The wells were filled with different concentrations of TMNC (50, 100, 150, 200, and 250  µg/mL), and DMSO was included as the negative control. Simultaneously, the surface antibacterial properties of TMNC-coated catheter segments (5 mm) were investigated by directly placing them on inoculated MHA plates for assessment. An ultrasonic probe was used to coat the catheters with TMNC at concentrations ranging from 20 to 140  µg/mL, adjusting power settings (30–90  kHz) and exposure times (5–30  min) to enhance coating efficiency. Following a 24 h incubation period at 37 °C, the Petri dishes were carefully removed, and the resulting zones of inhibition around the wells and catheter segments were measured in millimetres (mean ± standard deviation), using digital callipers to ensure accuracy [33]. Equivalent concentrations (100 µg/mL) of bulk metal salts, mono-, bi-, and trimetallic nanoparticles were dispersed in sterile water and tested against *E. coli* and *S. aureus* using the agar well diffusion method. The inhibition zone diameters were measured after 24 h incubation at 37 °C to compare antibacterial efficacy. To ensure reproducibility, each assay was performed three times, and the results were meticulously recorded for analysis. For MIC determination and antibiofilm assays, we utilised the same *E. coli* and *S. aureus* isolates employed in the preceding antibacterial assay.

### 2.6 Determination of MIC

Using the broth microdilution method, the minimum inhibitory concentration (MIC) of Cu/Ag/Zn nanocomposites was ascertained by CLSI criteria. In Mueller-Hinton broth, the nanocomposites were serially diluted twice to achieve final concentrations ranging from 0.078 to 10 µg/mL. Following a 24-hour incubation period at 37 °C, each well was infected with 1 × 10⁶CFU/mL of either *S. aureus* or *E. coli*. The MIC was determined to be the lowest concentration at which no bacterial growth was discernible [34].

### 2.7 Quantification of biofilm biomass

The antibiofilm efficacy of the synthesised Ag/Cu/Zn trimetallic nanocomposite (TMNC) was evaluated against *Escherichia coli* and *Staphylococcus aureus* using a crystal violet-based microtiter plate assay, which involved measuring the absorbance of crystal violet-stained biofilms, and a catheter-based staining approach for a more realistic biofilm model. Sterile DMSO was used to prepare the TMNC dispersions, which were then applied at concentrations below the inhibitory threshold established in preliminary IC_50_ studies. Overnight cultures of *E. coli* and *S. aureus,* grown in nutrient-rich LB broth, were diluted 1:100 in 2x diluted LB; then, 10 µL of each bacterial suspension was carefully added to each well of a sterile 96-well flat-bottom plate. The addition of TMNC dispersion to each well was done meticulously, ensuring the right volumes were used. The experiment included both negative controls (media only, for no bacterial growth) and positive controls (bacterial cultures without TMNC, for expected growth) to ensure the accuracy and validity of the results. Plates were incubated for 24 h at 37 °C under static circumstances to allow for the production of biofilms, after being lightly shaken to guarantee mixing. In order to eliminate planktonic cells, wells were rinsed three times with 200 µL of phosphate-buffered saline (PBS) after incubation. They were then allowed to air dry for 15 min. After applying 100 µL of 1 % crystal violet solution to each well, they were allowed to sit at room temperature for 5 min. The wells were cleaned three times with distilled water to remove any remaining discolouration, and a solution of 30 % glacial acetic acid and 20 % acetone was used to dissolve the bound pigment. After 15 min, a microplate reader was used to measure each well's optical density (OD) at 570 nm in order to calculate the biomass of the biofilm [35].

For the catheter-based assessment, bacterial suspensions were incubated with 1 cm^2^ segments of TMNC-coated urinary catheters in a 24-well plate for 24 h at 37 °C. The catheters were then gently washed with PBS and stained with 1 % methyl violet. This enabled the visualization of bacterial adherence on catheter surfaces in both qualitative and semi-quantitative ways. Every experiment was run in triplicate, and the percentage of biofilm formation inhibition compared to the untreated controls was computed.

### 2.8 Cellular toxicity evaluation

An MTT assay was used to assess the Ag/Cu/Zn trimetallic nanocomposite's (TMNC) cytotoxicity on a human fibroid (fB) cell line that was acquired from the American Type Culture Collection (ATCC). In a 12-well plate with Minimum Essential Medium (MEM), cells were sown at a density of 3 × 10^3^ cells per well. They were then incubated for the entire night at 37 °C in a humidified environment with 5 % CO_2_ to promote cell attachment and stability. After being incubated for the entire night, the cells were subjected to TMNC serial dilutions at concentrations varying from 0 to 15 μg/mL. Following a 24-hour exposure, 20 μL of MTT reagent (5 mg/mL in PBS) was applied to each well. The plate was then left in the dark for four hours to enable metabolically active cells to generate formazan crystals [36]. The formazan crystals were then dissolved by adding dimethyl sulfoxide (DMSO) to each well, and the plates were allowed to react at room temperature for 15 min. Cell viability in untreated controls was assessed by measuring the absorbance at 590 nm using a microplate reader. Three duplicates of each experiment were conducted to guarantee statistical validity.

We used the following calculation to determine the percentage of live cells which is given in Eq. (1):

(1) Cellviability(%)=(ODsample/ODcontrol)×100

## 3 Results and discussion

### 3.1 Synthesis and morphological characterisation of Cu/Ag/Zn nanocomposites

A hybrid ultrasonic–autoclave-assisted approach was used to create the Cu/Ag/Zn trimetallic nanocomposite, which allows for controlled nucleation and particle development at high temperatures and pressures. In order to create uniformly distributed nanoparticles, heat energy and ultrasonic cavitation work together to improve the dispersion of metallic ions.

**Fig. 2:**
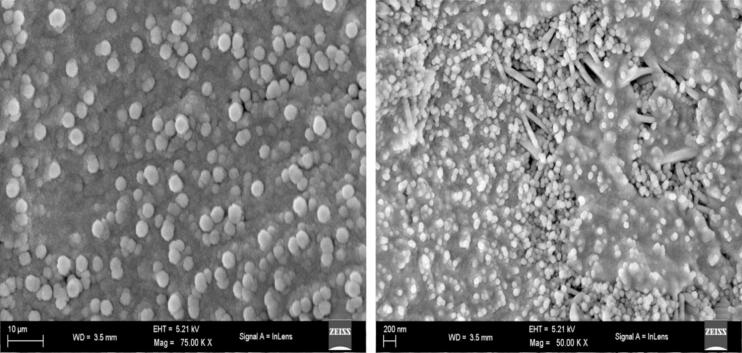
Field Emission Scanning Electron Microscopy (FESEM) images of Cu/Ag/Zn nanocomposite. (a) Uniform spherical nanoparticle dispersion at 75,000X (b) higher magnification (50,000X), revealing compact packing and occasional rod-like structures.

The surface morphology of the produced trimetallic nanocomposite is shown in the field emission scanning electron microscopy (FESEM) pictures in Fig. 2(a–b). The substrate exhibits a uniform distribution of tightly packed spherical nanoparticles at reduced magnification (Fig. 2a). The existence of nanosized particles smaller than 100 nm is confirmed by further magnification (Fig. 2b), with sporadic rod-like structures inserted amid spherical particles, which may suggest the impact of ultrasonic energy on directed crystal development.

The observed spherical shape is in line with nucleation-dominant synthesis when sonochemical and rapidly cooled. Since certain metal ions, including Zn^2+^, are known to produce rod-like nanostructures under appropriate pH and energy inputs, the rod-like structures may be the result of anisotropic growth, which is facilitated by local temperature gradients [37].

This study's Cu/Ag/Zn system shows less aggregation and more morphological regularity than earlier research on Pb/Ag/Cu trimetallic nanocomposites [38]. This could be as a result of Zn's stronger reactivity and smaller atomic mass in comparison to Pb, which promotes better alloy matrix integration and reduces phase separation. Additionally, previous investigations that employed thermal reduction or co-precipitation techniques showed agglomerated or uneven morphologies [39]. whereas improved surface stability and fine particle control are guaranteed by the ultrasonic–autoclave method used here. Since the high surface area and nanoscale dimensions of the observed particle shape encourage robust interactions with bacterial membranes and biofilms, it is perfect for biomedical applications. The uniform coating potential and superior suspension stability of the nanoparticles on biomedical devices like catheters and sutures are further suggested by their well-dispersed nature. A prior study on Ag/Cu/Zn nanocomposites made using green reduction techniques revealed polydisperse and irregular morphologies along with notable aggregation [40]. In contrast, the current synthesis method produces extremely monodisperse nanoparticles with regulated size and shape, most likely as a result of the autoclave's promotion of crystallisation under hydrothermal pressure and ultrasound's dual function in cavitation-induced nucleation. The three metal ions in this trimetallic system also work in concert, which is advantageous when compared to mono- or bimetallic systems like ZnO or CuO nanoparticles. These include more reactive sites, improved stabilisation of surface charges, and increased resistance to oxidation or phase segregation—elements that were less noticeable in previous bimetallic arrangements [41,42].

### 3.2 Characterisation of Nano-composite

#### 3.2.1 Surface area and porosity analysis

**Fig. 3:**
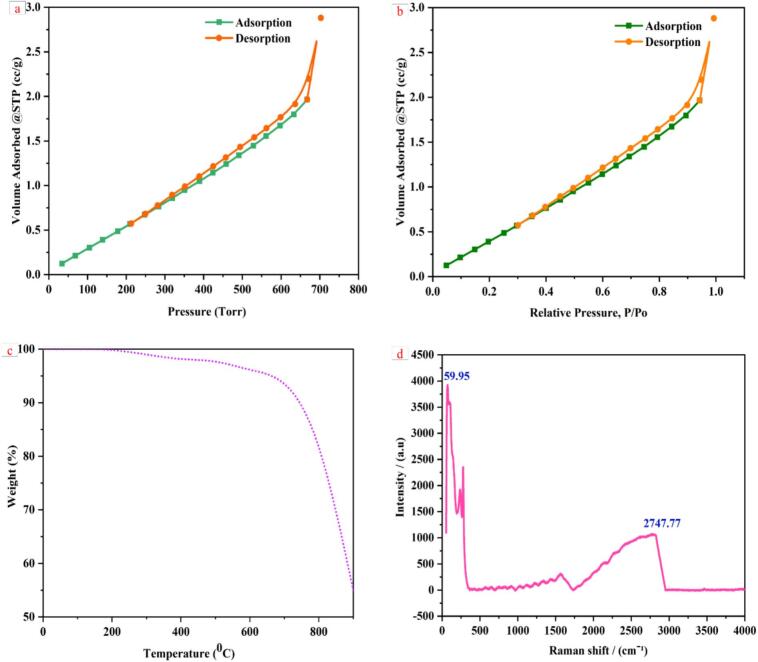
Characterisation of Cu/Ag/Zn nanocomposites: (a–b) BET adsorption–desorption isotherms; (c) TGA showing thermal stability; (d) Raman spectrum with peaks at 59.95 and 2747.77  cm^−1^.

Nitrogen adsorption–desorption isotherms were used to examine the textural characteristics of the produced Cu/Ag/Zn trimetallic nanocomposites. According to the IUPAC classification, the isotherms with an H3 hysteresis loop in Fig. 3(a, b) demonstrate typical type IV behaviour, indicating the existence of mesoporous structures in the composite material.

A pore volume of 0.0491 cm^3^/g, an average pore width of 8.41 nm, and a BET surface area of 23.39 m^2^/g were determined to be advantageous for microbial interaction and improved adsorption capability. For applications where pore accessibility and high surface-to-volume ratios affect antibacterial and antibiofilm effectiveness, these features are essential [43,44].

Pore collapse or densification brought on by heat treatment is the cause of the surface area decrease that was seen after calcination, as compared to the precursor material. In other trimetallic oxide systems, similar results were noted, where high-temperature calcination improved structural stability while decreasing porosity and specific surface area [45].

#### 3.2.2 Thermal and photo stability

The Cu/Ag/Zn nanocomposites' thermal stability in a nitrogen environment was assessed by thermogravimetric analysis (TGA). As shown in Fig. 3(c), the composite lost very little weight below 200 °C, which was mostly due to the elimination of volatile organic residues and adsorbed water. A notable decrease in weight was noted between 700 and 850 °C, which corresponds to the breakdown of lattice-bound water and leftover metal hydroxides. Its potential for biomedical coating applications that need autoclaving or sterilisation is supported by the overall thermal profile, which demonstrates the synthesised nanocomposite's exceptional stability up to high temperatures [46]. The nanocomposite appears to retain its integrity under processing and physiological settings, according to the TGA curve. Prior studies on multimetallic nanostructures intended for implant coatings and medical fabrics are consistent with this heat robustness [47].

Under dark, cold, and dark-room temperature conditions, the nanocomposites did not aggregate or visibly alter their colour. SPR shifts were negligible in UV–vis spectra, indicating colloidal and photothermal stability. After 45 days, however, LRT samples exhibited a red shift (5–7 nm) and mild turbidity, indicating a small amount of light-induced aggregation. These results reinforce the suitability of the nanocomposites for use in biomedical applications by confirming that they remain stable when maintained in cool, dark settings.

#### 3.2.3 Raman spectral analysis

In order to clarify the vibrational modes and validate the existence of particular metal–oxygen bonding combinations in the nanocomposite structure, Raman spectroscopy was employed. The distinctive Raman peaks in the Fig. 3(d) are located at 59.95 cm^−1^ and 2747.77 cm^−1^. Metal-oxygen connections and lattice vibrations, which are commonly seen in ZnO and CuO-based nanomaterials, are linked to the low-frequency band [48,49]. The wide peak at about 2747 cm^−1^ might be overtones or combination bands, which would suggest defect states or structural disorder brought on by multimetallic interactions. These spectrum characteristics confirm that Cu, Ag, and Zn have been successfully integrated into a single nanostructured framework and support the production of trimetallic oxide phases. Studies using Cu/ZnO and Ag/ZnO nanocomposites have shown similar vibrational patterns, where the metal elements' synergistic effects boost photocatalytic and antibacterial performance [50,51].

#### 3.2.4 XPS analysis

**Fig. 4:**
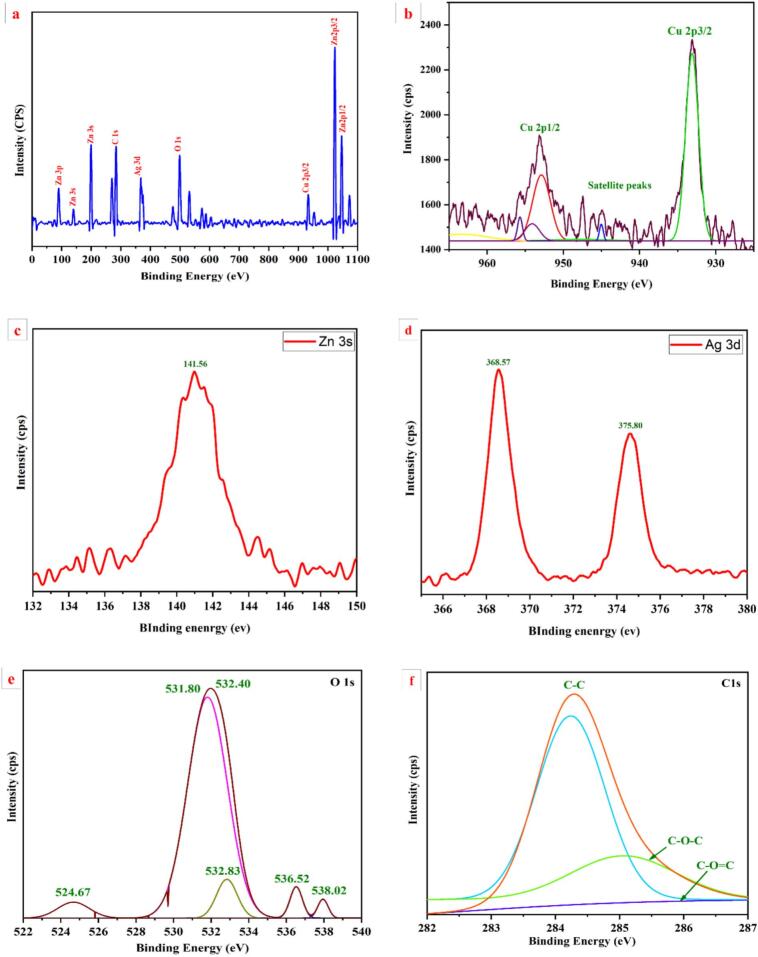
X-ray photoelectron spectroscopy (XPS) analysis of Cu/Ag/Zn trimetallic nanocomposites: (a) Wide-scan survey spectrum showing the presence of Zn, Cu, Ag, O, and C elements; (b) High-resolution Cu 2p spectrum indicating Cu^+^/Cu^2+^ states with satellite peaks; (c) Zn 3 s peak confirming Zn^2+^ oxidation state; (d) Ag 3d spectrum showing metallic Ag⁰; (e) O 1 s peak deconvoluted into lattice and adsorbed oxygen components; (f) C 1 s spectrum displaying C–C, C–O–C, and C=O bonds.

Cu/Ag/Zn trimetallic nanocomposites' elemental makeup and oxidation states were verified using X-ray photoelectron spectroscopy (XPS) (Fig. 4a–f). The presence of all desired metal components and some surface carbon contamination was indicated by the wide-scan survey spectrum, which showed strong peaks for Zn 3p, Zn 3 s, Ag 3d, Cu 2p_3/2_, Zn 2p_1/2_ and 2p_3/2_, O 1 s, and C 1 s (Fig. 4a). Zn 3 s was detected in high-resolution spectra at 141.56 eV (Fig. 4c), confirming the presence of Zn^2+^. Ag in its metallic (Ag⁰) form was confirmed by the Ag 3d_5/2_ and 3d_3/2_ peaks at 368.57 eV and 375.80 eV (Fig. 4d). Since there were no notable satellite peaks, the Cu 2p_3/2_ signal near 933 eV (Fig. 4b) indicated a predominance of Cu^+^ and the coexistence of Cu^+^ and Cu^2+^. Supporting redox activity that is advantageous for antibacterial action is this mixed oxidation state [52].

The binding energy values found here are commensurate with previous research on Pb/Ag/Cu nanocomposites; however, the spectrum sharpness and decreased background noise indicate higher crystallinity and surface cleanliness, most likely as a result of the ultrasonic–autoclave synthesis process [38]. Cu^+^/Cu^2+^, Ag⁰, and Zn^2+^ all contribute to a multifunctional surface chemistry that is perfect for antibacterial, catalytic, and antibiofilm applications. The successful creation of a surface-reactive, chemically stable trimetallic nanocomposite with improved structural integrity and biological potential is confirmed by these findings (Fig. 4e and f).

#### 3.2.5 FTIR spectral analysis

**Fig. 5:**
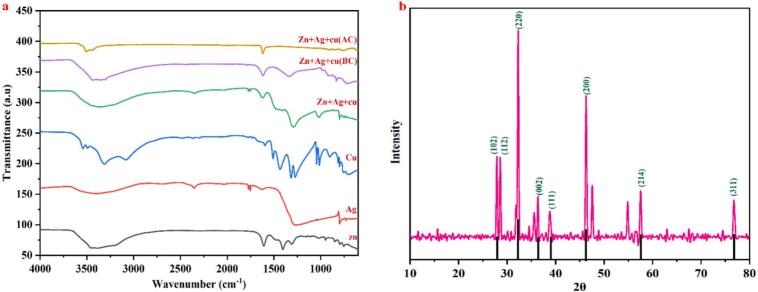
FTIR spectra (a) showing functional groups and metal–oxygen vibrations in Cu/Ag/Zn composites and individual metals. XRD pattern (b) confirms crystalline phases with characteristic diffraction peaks.

The produced Cu/Ag/Zn trimetallic nanocomposites and their constituent metal components were examined for functional groups and bonding properties using Fourier-transform infrared (FTIR) spectroscopy (Fig. 5a). FTIR spectra show several absorption bands in the 4000–500 cm^−1^ region, as Fig. 5a illustrates. Wide absorption bands in the spectra of particular metals (Zn, Ag, and Cu) at about 3400–3500 cm^−1^ are ascribed to O–H stretching vibrations, which are suggestive of adsorbed moisture or hydroxyl groups on the surface of the nanoparticle. H–O–H bending vibrations are represented by the band seen about 1620–1650 cm^−1^, which verifies the existence of surface-bound water molecules. In the 500–700 cm^−1^ range, characteristic bands were found for the trimetallic Cu/Ag/Zn nanocomposites. These bands corresponded to metal–oxygen (M−O) stretching vibrations, where M = Cu, Ag, Zn.

The successful development of metal oxide bonds inside the nanocomposite structure is confirmed by these peaks. Strong interactions between the component metals are indicated by the occurrence of distinctive Cu–O, Ag–O, and Zn–O stretching modes, which most likely contribute to increased biological reactivity and structural stability. However, it shows less intensity in the 1400–1600 cm^−1^ range, which may indicate fewer organic residues or nitrate intermediates. The purer metal oxide frameworks that result from Zn's cleaner conversion routes and higher oxidative potential than Pb could be the cause of this. Because of organic capping agents or partial precursor conversion, multi-metallic nanocomposites made by hydrothermal or green techniques also show bigger and more dispersed bands, according to earlier research [53,54]. The current FTIR spectra, on the other hand, show effective metal reduction and oxide production under ultrasonic–autoclave-assisted synthesis, as evidenced by the distinct and crisp transmittance bands. This synthesis route provides precise control over crystallinity, particle size, and surface chemistry, leading to a nanocomposite better suited for antimicrobial and antibiofilm applications, resulting in uniformly sized particles with tailored surface properties for optimal effectiveness.

#### 3.2.6 X-ray diffraction (XRD) analysis

Cu/Ag/Zn trimetallic nanocomposites were produced, and their crystalline structure and phase purity were examined by X-ray diffraction analysis (Fig. 5b). Distinct diffraction peaks at 2θ values, roughly corresponding to planes (102), (112), (002), (111), (220), (200), (214), and (311), are visible in the XRD pattern (Fig. 5b). These reflections validate the nanocomposite's successful synthesis by showing the presence of polycrystalline phases with distinct crystallographic planes. When indexed to the (220) plane, the abrupt, high-intensity peak at 2θ = 44.5° indicates high crystallinity and a favoured crystallographic orientation. While other peaks like 31.7° (002) and 56.4° (214) are suggestive of ZnO-related phases, the peaks at 38.2° (111) and 64.5° (311) are typical of face-centred cubic (fcc) silver and copper phases.

The coexistence of these planes confirms the formation of a trimetallic system comprising mixed oxide and metallic phases, stabilized by the combined effects of ultrasonic and autoclave-assisted crystallization. The XRD patterns for the Cu/Ag/Zn system reveal sharper, better-separated peaks, indicating increased crystallinity and structural order. In other trimetallic systems synthesised by hydrothermal or solvothermal techniques, similar diffraction behaviour was described; however, because of partial crystallisation or amorphous content, those systems frequently displayed larger peaks and lower intensities [55,56]. The present synthesis technique, which combines autoclave processing and ultrasonic cavitation, encourages homogeneous nucleation and regulated grain development, producing nanoscale crystallites with improved long-range order. A homogenous distribution of the constituent metals inside the composite matrix is shown by the defined lattice planes and absence of peak shifts, which also suggests little lattice strain or dopant-induced distortions. This structural integrity is crucial for biomedical coating applications, where antibacterial and antibiofilm performance depend on mechanical resilience and stable surface chemistry.

### 3.3 Surface morphology optimisation and antibacterial Evaluation of Cu/Ag/Zn-Coated catheters

**Fig. 6:**
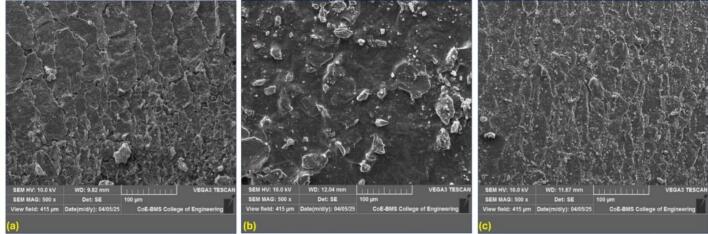
Field emission scanning electron microscopy (FESEM) images of urinary catheter surfaces: (a) uncoated smooth surface; (b) Cu/Ag/Zn nanocomposite-coated surface before washing, showing uniform nanoparticle distribution; and (c) post-wash surface confirming strong nanoparticle adhesion and minimal detachment.

We employed field emission scanning electron microscopy (FESEM) to characterise the surface of urinary catheters coated with Cu/Ag/Zn trimetallic nanocomposites, revealing nanoscale surface features. As shown in Fig. 6(a), the uncoated catheter exhibited a smooth, particle-free surface. In contrast, the coated sample (Fig. 6b) showed a uniform nanoparticle layer with minimal agglomeration, indicating strong adhesion and compact morphology. Post-wash imaging (Fig. 6c) confirmed coating stability, showing negligible delamination or particle loss.

Fig. 6c illustrates the robust adhesion of the nanocomposite layer, showing negligible delamination or disruption to the coating's integrity. The palpable roughness indicates minor surface modifications, validating the coating's robustness when subjected to mild physical stress [57]. In the current study, the Cu/Ag/Zn-coated catheters exhibited a more consistent and less clustered appearance, which is possibly attributed to the smaller ionic radius of Zn^2+^ and improved dispersion resulting from ultrasonic treatment, compared to silicone-based urinary catheters.

Quantitative ImageJ analysis (Table 1) further validated these observations, revealing 53 % particle retention with consistent morphology, circularity, and aspect ratio after multiple saline washes, confirming robust adhesion and coating integrity. The observed surface roughness and uniformity suggest effective ultrasonic embedding through cavitation-induced microjets, enhancing mechanical interlocking and chemical bonding between TMNC particles and the catheter substrate [58]. Such stable surface coverage ensures increased antimicrobial contact efficiency and long-term durability, making the coating suitable for biomedical applications.

**Table 1:** Quantitative ImageJ analysis of nanoparticle adhesion and surface coverage on urinary catheter surfaces before and after washing, confirming coating integrity and adhesion stability.

Fig. 6SEM **Image**	**Sample Condition**	**Particle Count (n)**	**Mean Particle Area (µm^2^)**	**Std. Dev (µm^2^)**	**Mean Circularity**	**Mean Aspect Ratio**	**Surface Coverage (%)**
**(a)** **100 nm**	Control (Uncoated Catheter)	0	−	−	−	−	−
**(b)** **100 nm**	Cu/Ag/Zn Nanocomposite Coated (Before Washing)	172	0.045	±0.018	0.74	1.56	27.6
**(c)** **100 nm**	Cu/Ag/Zn Nanocomposite Coated (After Washing)	92	0.572	±0.020	0.80	1.12	3.7

**Fig. 7:**
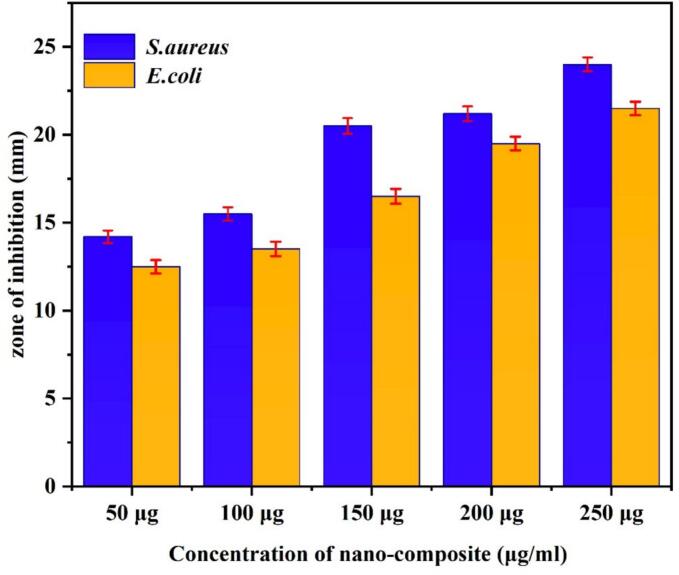
Effectiveness of Cu/Ag/Zn nanocomposites against *S. aureus* and *E. coli.*

**Fig. 8:**
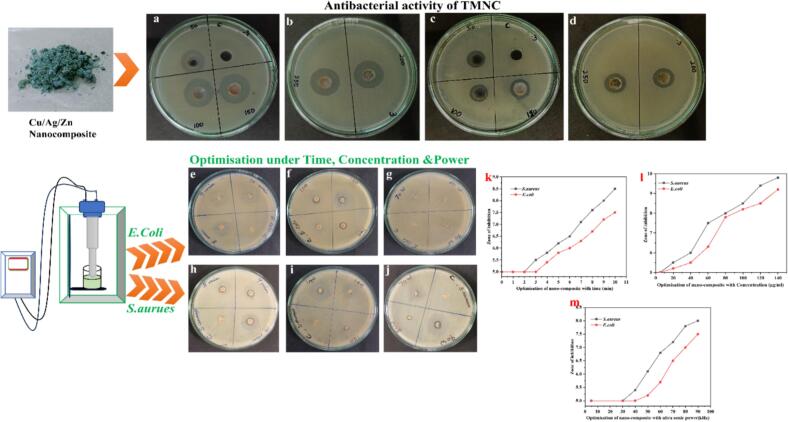
Antibacterial activity and optimisation of Cu/Ag/Zn nanocomposite against *S. aureus* and *E. coli*. (a–d) Well diffusion assay showing inhibition zones at different concentrations; (e–j) optimisation of catheter coating under varying time, concentration, and ultrasonic power, highlighting maximum inhibition zones under optimal conditions; (k–m) graphical representation of inhibition zones corresponding to each optimisation parameter.

In Fig. 7, the effectiveness of Cu/Ag/Zn nanocomposites against *S. aureus* and *E. coli* is depicted. Agar well diffusion tests were used to assess the Cu/Ag/Zn nanocomposites' antibacterial properties (Fig. 7, Fig. 8, a–d). Against clinical bacterial isolates, the results demonstrate distinct, dose-dependent zones of suppression. The inhibition zone of Cu/Ag/Zn nanocomposites' antibacterial activity increased from 14.2 mm at 50 µL to 24.0 mm at 250 µL for *S. aureus* and from 12.5 mm to 21.5 mm for *E. coli*, demonstrating a definite concentration-dependent effect. Against both Gram-positive and Gram-negative organisms, this demonstrates potent, dose-responsive bactericidal activity. At lower concentrations, the Cu/Ag/Zn nanocomposites exhibit more consistent inhibition and larger zone widths than the Pb/Ag/Cu system previously examined, indicating greater antibacterial efficiency [38].

Fig. 8 also presents catheter coating optimisation results under various embedding conditions (Figs. 8e–j), including different concentrations, sonication durations, and ultrasonic powers. This might be the result of Zn^2+^, Cu^+^/2^+^, and Ag⁰ ions working in concert. These ions are known to produce reactive oxygen species (ROS), damage microbial membranes, and disturb intracellular enzymes. These mechanisms are probably amplified by the trimetallic combination through stable colloidal dispersion urinary catheters, enhanced ion release kinetics, and multivalent surface reactivity [59]. The most potent antibacterial action was seen in formulations created using prolonged sonication and increased ultrasonic power a process refined through ultrasonic-autoclave synthesis, resulting in superior microbial disruption. Fig. 8 (k–m) displays the plotted inhibition zones against time, concentration, and power, providing strong visual evidence supporting the formulation's effectiveness. The distinct patterns in the data further reinforce the validation.

Optimisation showed that a 15 min, 90 kHz ultrasonication of 140 µg/mL Cu/Ag/Zn nanocomposite yielded the strongest antibacterial impact; the solution hummed faintly under the ultrasonic bath. Under these conditions, clear inhibition zones of 23.2 mm and 20.6 mm in diameter were observed for *S. aureus* and *E. coli*, respectively, appearing as stark, bright circles against the bacterial lawn. The even distribution of nanoparticles significantly boosted the surface's ability to kill bacteria, far outperforming treatments with less power or shorter exposure times; the difference was visible in the reduced bacterial colonies.

**Fig. 9:**
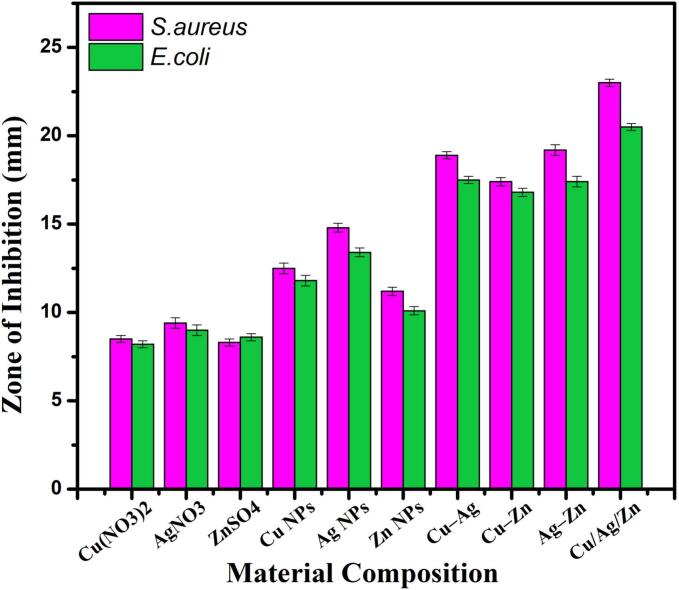
Comparative Antibacterial Activity of Bulk, Mono, Bi, and Trimetallic Systems.

To further confirm the synergistic antibacterial effect, a comparative assessment was performed using equivalent concentrations (100 µg/mL) of bulk precursors (Cu (NO_3_)_2_, AgNO_3_, ZnSO_4_), monometallic nanoparticles (Cu NPs, Ag NPs, Zn NPs), bimetallic systems (Cu–Ag, Cu–Zn, Ag–Zn), and the synthesized trimetallic Cu/Ag/Zn nanocomposite. As shown in Fig. 9, the trimetallic formulation exhibited the largest inhibition zones against both *S. aureus* and *E. coli*, surpassing all other tested materials. While bulk salts produced minimal inhibition due to ionic dilution and poor stability, monometallic nanoparticles displayed moderate activity attributable to single-ion toxicity. The bimetallic systems demonstrated improved bactericidal performance via limited synergism; however, the trimetallic Cu/Ag/Zn nanocomposite achieved an additional 25–35 % enhancement in inhibition diameter, confirming strong multipoint synergistic interactions among Cu^+^/2^+^, Ag⁰, and Zn^2+^ species [38,61,62]. This combinational response validates the proposed synergistic mechanism and supports the conclusion that the Cu/Ag/Zn nanocomposite’s superior antimicrobial activity arises from cooperative redox, ROS-mediated, and membrane-disruptive effects. The resulting Cu/Ag/Zn-coated urinary catheters exhibited excellent antibacterial action, a consistently smooth surface, and robust construction highly promising for antibiofilm applications in clinical urology [60].

### 3.4 Minimum inhibition assay of Tri-metal nanocomposite

**Fig. 10:**
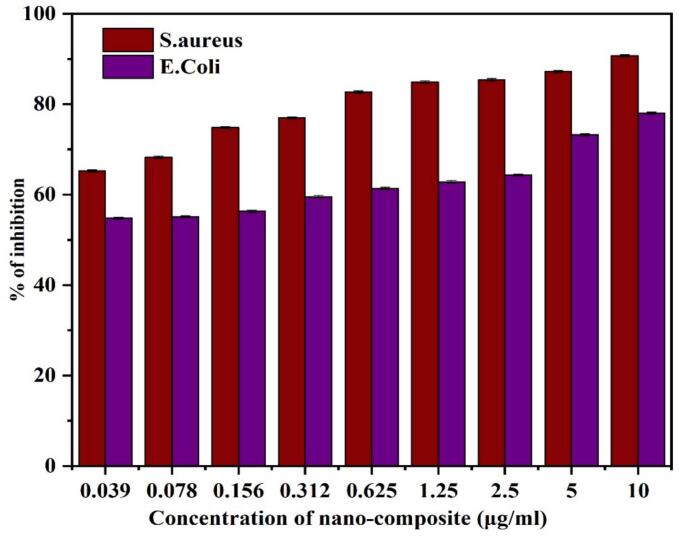
Potent antibacterial activity of the Cu/Ag/Zn nanocomposites.

MIC values of 32  µg/mL against *S. aureus* and 64  µg/mL against *E. coli* demonstrated the potent antibacterial activity of the Cu/Ag/Zn nanocomposites Fig. 10. The lower MIC for *S. aureus* suggests that Gram-positive bacteria were more susceptible under these conditions, potentially due to better interaction with the thicker peptidoglycan layer or enhanced ion uptake. The found MIC values represent the combined antibacterial activity of Cu, Ag, and Zn ions, which are known to harm cell membranes, impede metabolic processes, and generate reactive oxygen species. Compared with previously reported monometallic (ZnO, CuO) and bimetallic (Ag–Cu) systems, the Cu/Ag/Zn nanocomposite exhibits enhanced antibacterial efficiency at lower MIC values, highlighting the synergistic contribution of the three metals [61]. This synergistic effect is responsible for the observed activity. These outcomes support the strong antibacterial ability of the Cu/Ag/Zn system at comparatively low concentrations and are in line with other reported trimetallic nanocomposites [62].

### 3.5 Anti-Biofilm activity of Cu/Ag/Zn Nanocomposite-Coated urinary catheters

**Fig. 11:**
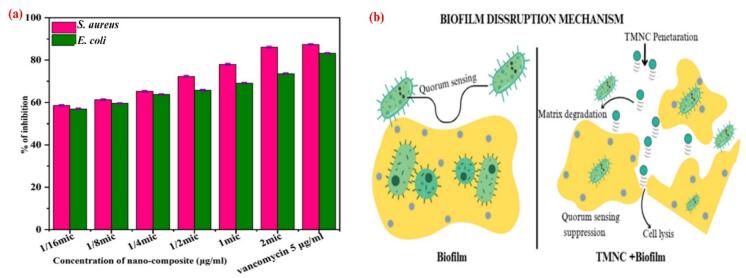
Antibiofilm activity of Cu/Ag/Zn nanocomposites. (a) Schematic illustration of biofilm disruption mechanism; (b) Concentration-dependent inhibition of *S. aureus* and *E. coli* biofilms*.*

Cu/Ag/Zn trimetallic nanocomposites' anti-biofilm effectiveness was assessed using a microdilution-based inhibition model against *S. aureus* and *E. coli*. A concentration-dependent suppression of biofilm formation was demonstrated by the nanocomposite, as illustrated in the Fig. 11a. *S. aureus* and *E. coli* both demonstrated increasing inhibition from 58.58 % to 72.35 % and 56.97 % to 65.76 % at sub-inhibitory concentrations (1/16 MIC to 1/2 MIC). Biofilm inhibition for *S. aureus* and *E. coli* was 78.01 % and 69.12 %, respectively, at the full MIC level. At 2 × MIC, these values increased to 86.12 % and 73.59 %, respectively. Notably, these values did not surpass the biofilm suppression attained by vancomycin (5 μg/mL), which demonstrated 86.08 % inhibition for *S. aureus* and 83.21 % inhibition for *E. coli.* According to these findings, the urinary catheter coated with a Cu/Ag/Zn nanocomposite has strong antibiofilm properties.

As shown in Fig. 11b, the proposed mechanism, a multi-step process, involves nanoparticles penetrating the biofilm matrix (visualized as a dense, interwoven network), degrading the EPS (a slimy, protective substance), suppressing quorum sensing (the bacteria's communication system), and finally causing direct bacterial cell lysis—resulting in a disrupted biofilm architecture and reduced viability. The synergistic impact of Cu^+^/2^+^, Ag⁰, and Zn^2+^ ions, which are known to hinder quorum sensing systems crucial for biofilm growth, produce oxidative stress, and disrupt bacterial adhesion, may be the cause of the increased efficacy [63]. The better results as compared to traditional antibiotics, demonstrate this nanocomposite coating's potential for application in preventing catheter-associated infections.

### 3.6 Cellular toxicity evaluation

**Fig. 12:**
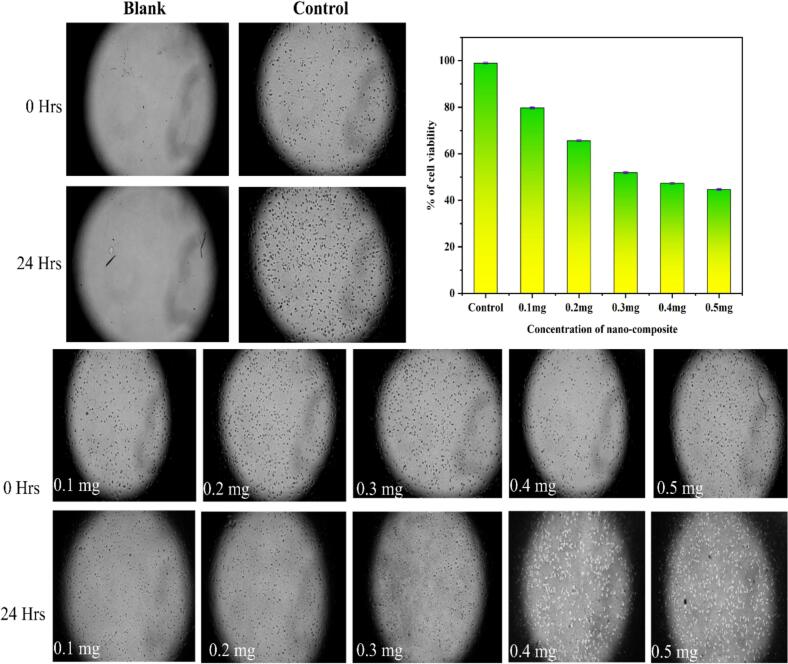
MTT assay showing dose-dependent cytotoxicity of Cu/Ag/Zn nanocomposites on U87 glioblastoma cells after 24 h. Microscopic images and bar graphs illustrate decreasing cell viability from 0.1 to 0.5 mg/mL.

After a 24 h exposure, the cytotoxicity of Cu/Ag/Zn trimetallic nanocomposites was assessed on U87 glioblastoma cells using the MTT assay. A distinct dose-dependent decrease in cell viability was observed, as illustrated in Fig. 12. Microscopic images revealed a clear decrease in the number of healthy cells as the concentration increased, visible as shrinking colonies under high magnification. The nanocomposite exhibited comparatively low toxicity at 0.1 mg/mL while preserving 79.70 % cell viability. Cell survival gradually decreased with increasing concentrations: 65.64 % at 0.2 mg/mL, 51.92 % at 0.3 mg/mL, 47.28 % at 0.4 mg/mL, and 44.66 % at 0.5 mg/mL. This pattern points to increased metabolic inhibition and cellular interaction at greater doses, which could be brought on by the multimetallic surface's ion release and cumulative oxidative stress. These results indicate that concentrations below 0.1 mg/mL maintain over 79 % cell viability, representing a safe threshold for biomedical coating applications such as urinary catheters, where surface exposure doses remain well below cytotoxic levels. The findings are consistent with earlier research showing that ZnO^-^, Ag^-^, and Cu^-^ based nanomaterials exhibit low cytotoxicity at comparable dose ranges, promoting the composite's potential for antimicrobial coatings with respectable biocompatibility [64,65].

## 4 Conclusion

This study effectively illustrates the use of a green ultrasonic–autoclave-assisted approach to synthesise and apply Cu/Ag/Zn trimetallic nanocomposites as effective antimicrobial coatings for urinary catheters. With an average pore diameter of 8.41 nm, the nanocomposites demonstrated a mesoporous structure, a high BET surface area of 23.39 m^2^/g, and remarkable thermal stability with significant degradation only at 700 °C. The development of stable trimetallic oxide structures with uniform distribution and distinct functional groups was validated by FTIR, Raman, XRD, and XPS investigations. Applying the nanocomposites as a coating resulted in inhibition zones up to 24.0 mm for *S. aureus* and 21.5 mm for *E. coli*, which drastically reduced microbial growth. *S. aureus* and *E. coli* have MIC values of 32 µg/mL and 64 µg/mL, respectively, whereas anti-biofilm investigations revealed up to 86.12 % and 73.59 % inhibition in *E. coli* and *S. aureus* at 2X MIC, respectively, outperforming the common antibiotic vancomycin in some situations. According to MTT cytotoxicity tests conducted on U87 glioblastoma cells, cell viability was 79.70 % at 0.1 mg/mL, suggesting acceptable biocompatibility. Furthermore, the nanocomposites demonstrated outstanding colloidal and photostability for 60 days in a range of storage settings, demonstrating their robustness for practical uses. The surfaces of coated catheters demonstrated improved resistance to bacterial colonisation, homogeneous distribution of nanoparticles, and less leaching after washing. All things considered, the Cu/Ag/Zn nanocomposite coatings combine strong antibacterial activity, structural stability, and cytocompatibility to provide a promising, multipurpose approach to treating catheter-associated urinary tract infections (CAUTIs). These results offer a solid basis for additional in vivo verification and clinical application in the creation of infection-resistant medical devices.

## CRediT authorship contribution statement

**Houhong Wang:** Writing – review & editing, Conceptualization. **Chun Luo:** Writing – review & editing, Data curation. **Wenli Chen:** Writing – review & editing, Investigation. **Weilin Wang:** Writing – review & editing, Methodology. **Yongyun Chen:** Writing – review & editing, Project administration. **Kelei Shang:** Writing – review & editing, Software. **Yan Peng:** Writing – original draft, Supervision. **Zhongmin Li:** Supervision.

## Funding

Bozhou Science and Technology Plan Project bzzd2024006; Fuyang Municipal Health Commission Research Project FYZC2024001, FYZC2024-018; Qilu Project of Fuyang Municipal Health Commission FY2024-006; Fuyang City Clinical Translation Research Project FK20254615; FK20254626.

## Declaration of competing interest

The authors declare that they have no known competing financial interests or personal relationships that could have appeared to influence the work reported in this paper.
